# Common-Mode Noise Reduction in Noncontact Biopotential Acquisition Circuit Based on Imbalance Cancellation of Electrode-Body Impedance

**DOI:** 10.3390/s20247140

**Published:** 2020-12-13

**Authors:** Minghui Chen, Jianqing Wang, Daisuke Anzai, Georg Fischer, Jens Kirchner

**Affiliations:** 1 Department of Electrical and Mechanical Engineering, Nagoya Institute of Technology, Gokiso-cho, Showa-ku, Nagoya 466-8555, Japan; m.chen.032@stn.nitech.ac.jp (M.C.); Anzai@nitech.ac.jp (D.A.); 2 Institute for Electronics Engineering, Friedrich-Alexander-Universität of Erlangen-Nuremberg, Schlossplatz 4, 91054 Erlangen, Germany; georg.fischer@fau.de (G.F.); jens.kirchner@fau.de (J.K.)

**Keywords:** biomedical electronics, common-mode noise, electromagnetic interference, noncontact electrode

## Abstract

Biopotential sensing technology with electrodes has a great future in medical treatment and human—machine interface, whereas comfort and longevity are two significant problems during usage. Noncontact electrode is a promising alternative to achieve more comfortable and long term biopotential signal recordings than contact electrode. However, it could pick up a significantly higher level of common-mode (CM) noise, which is hardly solved with passive filtering. The impedance imbalance at the electrode-body interface is a limiting factor of this problem, which reduces the common mode rejection ratio (CMRR) of the amplifier. In this work, we firstly present two novel CM noise reduction circuit designs. The circuit designs are based on electrode-body impedance imbalance cancellation. We perform circuit analysis and circuit simulations to explain the principles of the two circuits, both of which showed effectiveness in CM noise rejection. Secondly, we proposed a practical approach to detect and monitor the electrode-body impedance imbalance change. Compared with the conventional approach, it has certain advantages in interference immunity, and good linearity for capacitance. Lastly, we show experimental evaluation results on one of the designs we proposed. The results indicated the validity and feasibility of the approach.

## 1. Introduction

As the aging society problem draws great attention, the sensing technology of biosignals, especially biopotential signals like electrocardiogram (ECG), electroencephalogram (EEG) or electrooculogram (EOG), has advanced tremendously over the years. As an example, the body area network (BAN) with wearable sensing technologies [1,2,3] collects vital data for health-state monitoring, which is considered as an emerging solution to soaring healthcare costs and shortages of medical resources.

In addition to medical treatment and healthcare, the biosignals could also be considered as a significant interface between human body and machines. In the literature, applications like activity recognition [4], driving assistance [5,6] or human-computer interface [7,8,9] were mentioned by researchers. The potential of biosignals is still to be exploited when sensing technologies advance further. Comfort, longevity and precision are three main topics about the advancements of electrode-based sensing technology.

Wet electrode is the most common type for both clinical and research applications. A typical wet electrode consists of a silver-silver chloride metal that is surrounded by a wet or solid hydrogel, containing chloride. The primary drawbacks with wet electrodes are limited lifetime, discomfort and skin irritations. Wet electrodes degrade as the moisture content evaporates, limiting its useful lifetime to several hours, or at most a few days. The oxidation processes may also have some influence, but normally not mentioned as the main factor. Dry contact electrodes operate without the use of wet/gel coupling media. The metal in the electrode directly contacts the skin to couple biopotential signals. However, dry contact electrodes still rely on some degree of moisture. The performance of a dry contact electrode usually increases over time as more moisture permeates the skin-electrode interface.

The final type of electrodes, noncontact, can be thought of as a special case of dry electrodes. The noncontact electrode was introduced by Lopez and Richardson in 1967 [10] and further developed in the last decade [11,12,13,14]. Noncontact electrodes have certain advantages in biosignal acquisition because they do not need direct skin contact, and they can be integrated in objects like beds or cars for long-term biosignal recording. They operate not only without gel, but also through an insulation layer such as clothing. Therefore, they are also called capacitive electrodes. There are also some other technologies that can achieve noncontact sensing of biosignals, like using the Doppler cardiogram [15] or a radar [16,17,18,19]. Recently, Taylor et al. [20] provided a noncontact approach to detect COVID-19.

The coupling capacitance of a noncontact electrode can range in the order of tens or hundreds of picofarad [21]. Obtaining acceptable signals requires the use of high input impedance active electrodes. Impedance at the electrode-body interface (EBI) of noncontact electrodes are highly sensitive to environmental conditions such as humidity and the exact insulating material. This property makes the noncontact electrodes induce much more noise than wet electrodes, because the imbalance of the EBI impedances allows the common-mode (CM) noise converting into a differential mode (DM) interference voltage [22]. Liao et al. [23] explained to what extent an imbalance in the impedances of EBI could reduce the common mode rejection ratio (CMRR). As a result, CM noise source like the power line, electromagnetic interference (EMI) or a wireless power transfer system [23] can severely interfere with the detection progress. Moreover, because of this, a body or skin motion could change the coupling conditions such that the imbalance varies and induces more noise; cancelling the imbalance can also reduce motion artefacts thanks to improving balance, thus raising CMRR.

There are also imbalances in other circuit elements. Perfect circuit elements for complete impedance matching in sensing circuits is not a practical issue in the industry. Because the biopotentials are generally at very low voltage level (microvolts or millivolts) and almost the same frequency (0 to 1 kHz) as the noise, a small deviation of impedance value may lead to a noise at millivolts in worst case. The well-known instrumentation amplifier (INA) uses two amplifying buffers and a bridging resistor to restore the CMRR of a differential amplifier, which is a common strategy for biopotential acquisition nowadays. However, for the case of noncontact electrodes, it is not possible to acquire high quality biosignals only by using an INA, especially when there is a body or skin motion. Chi et al. [24] expressed the CMRR in the following shape:

(1) $$CMRR\approx \frac{Z_{i}}{|Z_{1}-Z_{2}|}$$

where $Z_{1}$ and $Z_{2}$ are the EBI impedances, $Z_{i}$ is the input impedance of the biopotential amplifier. At low frequencies like 50/60 Hz, the impedances of noncontact EBIs are primarily capacitive and the expression simplifies to

(2) $$CMRR\approx \frac{C_{1}C_{2}}{C_{i}\left|C_{1}-C_{2}\right|}$$

From (2), we can see that for noncontact/capacitive electrodes, an imbalance of picofarads between the electrodes can reduce the CMRR more severely than contact ones, whose imbalance is usually in the order of kiloohms. This fact is the reason why a capacitive sensing scheme is so sensitive to imbalance variations.

In our previous work [25], we presented the principle of a CM noise reduction approach for contact biopotential acquisition. Our work on contact electrodes basically shares the same principles with this work in cancelling the imbalance of EBI. A pair of digital potentiometers (DigiPOTs) were employed to compensate the imbalance of EBI resistance. DigiPOTs has a certain advantage in cancelling the imbalance of resistance in the EBI of contact electrodes, because they can be tuned exactly and conveniently with a digital control signal. However, when it comes to noncontact electrodes, it will not work as successfully as for the contact electrodes, because the imbalance becomes capacitive. Serteyn et al. [26] proposed an approach to exploit an injection signal to track the capacitance change of EBI and restore the ECG signal through digital processing. Their approach taught us how to monitor the imbalance change at the EBI, but their work did not solve the imbalances caused by the other circuit elements.

In this work, we propose using digitally tunable capacitor (DTC) to cancel the imbalance of EBI capacitance. A DTC is an IC variable capacitor that can be controlled with a digital signal. We also use an injection signal to monitor the variance of EBI capacitance, whereas the circuit design is completely original with a microcomputer to control the DTC and reject the CM noise, which could also work on the imbalance of the other circuit elements, as we mentioned. As in [25], we use a pseudo EMI noise source in simulation evaluation to quantitatively show the effectiveness of our approach. We also use circuit boards to confirm its validity and feasibility in a laboratory environment.

## 2. Circuit Analysis

### 2.1. Proposal 1: “DTC Series”

**Figure 1:**
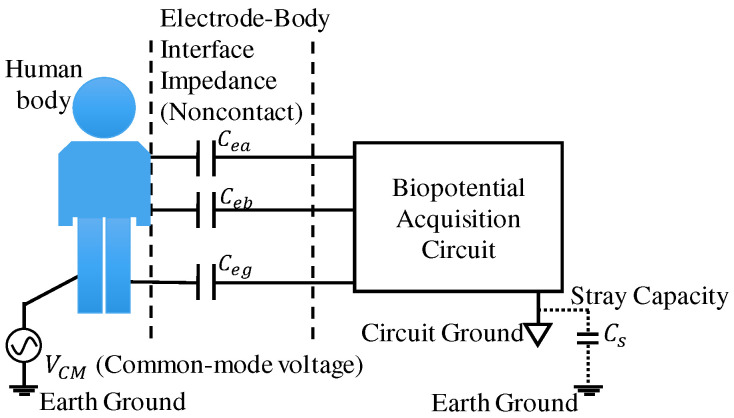
Assumed acquisition model and conditions of circuit analysis.

In this section, we present our biopotential acquisition circuit design proposals and analyze how they work in reducing the CM noise. The assumed acquisition model and the conditions of our circuit analysis are shown in Figure 1. There are two noncontact sensing electrodes and one ground electrode. The biopotential signals are acquired from the two sensing electrodes. The EBI capacitance is denoted as $C_{ea}$ or $C_{eb}$. Usually, they are unbalanced due to the different attachment conditions. $C_{eg}$ denotes the impedance of the ground electrode. In addition, the stray capacity between the circuit ground and the earth ground is denoted as $C_{s}$. The interference voltage $V_{CM}$ is between the human body and the earth ground. This model could be considered as a usual one for single channel ECG, EEG, EOG, etc. Theoretically, a 2-wired sensing circuit can also be employed to acquire one channel of biopotential, but a ground electrode or a driven-right-leg (DRL) electrode is generally necessary to reject noise and reduce errors.

**Figure 2:**
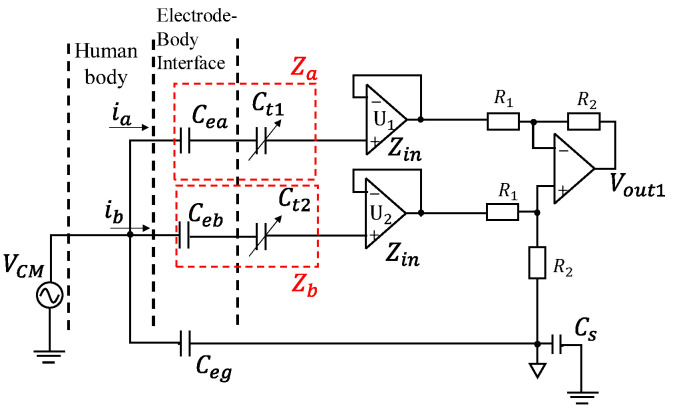
Common-mode (CM) equivalent circuit of digitally tunable capacitor series (“DTC Series”).

Figure 2 shows the CM equivalent circuit of our first proposal, which we call “DTC Series”. It is based on the noncontact 3-wired single channel biopotential acquisition model shown in Figure 1. Two buffer amplifiers are used to provide high input impedance. There are two grounds for the circuits in Figure 2 and the other circuits we handle. One is the circuit ground for differential mode voltage. The operational amplifier (OP-AMP)’s ground is this ground and the OP-AMP circuit thus works normally. The other ground is the earth ground for common mode voltage. This ground is floating, and the floating is difficult to be solved by autozero or a similar mechanism because it is the earth. The input to the OP-AMP contains both the differential mode voltage and the common mode voltage, and the common mode voltage is converted into a differential voltage as a noise at the OP-AMP output. Between the electrodes and the buffers, two DTC elements, $C_{t1}$ and $C_{t2}$, are installed in series to the input impedance of the buffer. The biopotential signals are differentially amplified with an OP-AMP. The OP-AMPs are not ideal because if they were, the currents $i_{a}$ and $i_{b}$ in Figure 2 would become zero and the CM noise would not affect this circuit. The input offset voltages of the buffers are designed to be very low, such that the input and output voltages of them are assumed to be equal. With the conditions that we gave, $V_{out1}$ can be written as

(3) $$V_{out1}=\frac{j\omega CZ_{eg}R_{1}\left(R_{1}+R_{2}\right)\left(Z_{eg}+Z_{in}\right)\left(Z_{a}-Z_{b}\right)V_{CM}}{R_{2}\left(Z_{ea}+Z_{in}\right)\left[\left(1+j\omega CZ_{eg}\right)\left(R_{1}+R_{2}\right)\left(Z_{eg}+Z_{in}\right)+Z_{eg}\left(Z_{a}-Z_{b}+Z_{in}\right)\right]}$$

where

(4) $$Z_{a}-Z_{b}=\left(\frac{C_{ea}-C_{eb}}{j\omega C_{ea}C_{eb}}+\frac{C_{t1}-C_{t2}}{j\omega C_{t1}C_{t2}}\right)$$

$Z_{in}$ is the input impedance (with respect to the circuit ground) of the amplifier U1 or U2 (buffers). From (3), we can know that if ($Z_{a}$−$Z_{b}$) equals zero, then the output of CM noise $V_{out}$ also becomes zero. Therefore, we can adjust the capacitance of $C_{t1}$ and $C_{t2}$ to cancel the imbalance between $C_{ea}$ and $C_{eb}$, such that the CM noise is reduced. Besides, this imbalance cancellation is independent of the frequency, which means that theoretically we can drive down the CM noise of all frequencies to extremely low level once $C_{t1}$ and $C_{t2}$ are exactly adjusted.

### 2.2. Proposal 2: “DTC Bypath”

**Figure 3:**
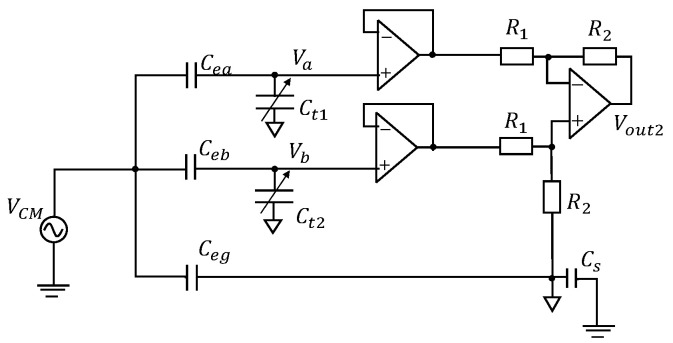
CM equivalent circuit of “DTC Bypath”.

There is another circuit design to achieve capacitive imbalance cancellation. Its CM equivalent circuit is shown in Figure 3. This design tries to make the voltage at $V_{a}$ and $V_{b}$ equal, like the famous Wheatstone bridge. We call this proposal the “DTC Bypath”. In this circuit, $V_{out2}$ can be written as

(5) $$V_{out2}=\Psi \cdot \frac{\Gamma }{\Pi }\cdot V_{CM}$$

where

(6) $$\Gamma =\omega^{2}Z_{in}\left(C_{ea}C_{t2}-C_{eb}C_{t1}\right)+j\omega \left(C_{eb}-C_{ea}\right)$$

(7) $$\Psi =\frac{j\omega C_{s}\left(R_{1}+R_{2}\right)\left[j\omega \left(C_{eb}+C_{t2}\right)Z_{in}+1\right]}{R_{2}j\omega (C_{eb}Z_{in}+j\omega \left(C_{eg}+C_{s}\right)\left[j\omega \left(C_{eb}+C_{t2}\right)Z_{in}+1\right]\left(R_{1}+R_{2}\right)}$$

(8) $$\Pi =-\omega^{2}Z_{in}^{2}\left(C_{eb}+C_{t2}\right)\left(C_{ea}+C_{t1}\right)+j\omega \left(C_{ea}+C_{t1}+C_{eb}+C_{t2}\right)$$

Ψ and Π are coefficients given by (7) and (8). If we want $V_{out2}$ to be zero, which can be derived by letting Γ = 0, we can obtain

(9) $$\frac{C_{ea}}{C_{eb}}=\frac{j\omega C_{t1}Z_{in}+1}{j\omega C_{t2}Z_{in}+1}$$

This equation can also be derived by letting $V_{a}$ = $V_{b}$, where $V_{a}$ and $V_{b}$ are the input voltages of the buffers denoted in Figure 3. From this equation, we can see that the CM noise can be reduced by adjusting the $C_{t1}$ and $C_{t2}$. It should be noted that (9) is affected by frequency, and if $\omega Z_{in}$ is large enough, (9) becomes

(10) $$\frac{C_{ea}}{C_{eb}}=\frac{C_{t1}}{C_{t2}}$$

It reveals that by employing “DTC Bypath”, we can reduce the CM noise through matching $C_{ea}$/ $C_{eb}$ and $C_{t1}$/$C_{t2}$.

## 3. Circuit Simulation and Results

### 3.1. Without the DTCs

In this section, we use the SPICE (simulation program with integrated circuit emphasis) to show simulation results of our proposal, in comparison with a circuit without the DTCs.

**Figure 4:**
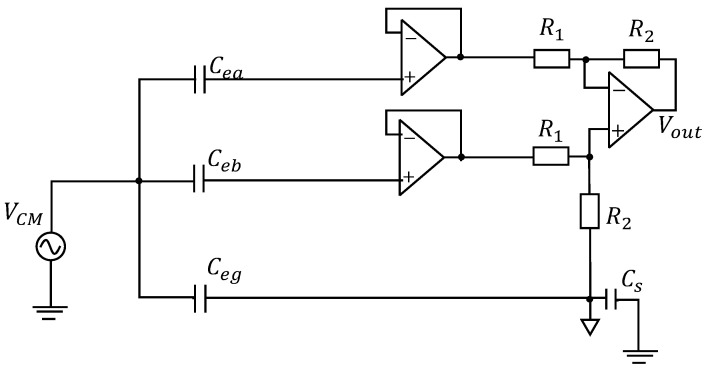
CM equivalent circuit without the DTCs.

Figure 4 shows the CM equivalent circuit for biopotential acquisition without the DTCs. Parameters of the simulated circuit components are shown in Table 1. All the OP-AMPs in the circuit are simulated with the model of OP07 (analog devices), because it has very low input offset voltage, low input bias current and high open-loop gain, which make it suitable for high gain instrumental applications.

**Figure 5:**
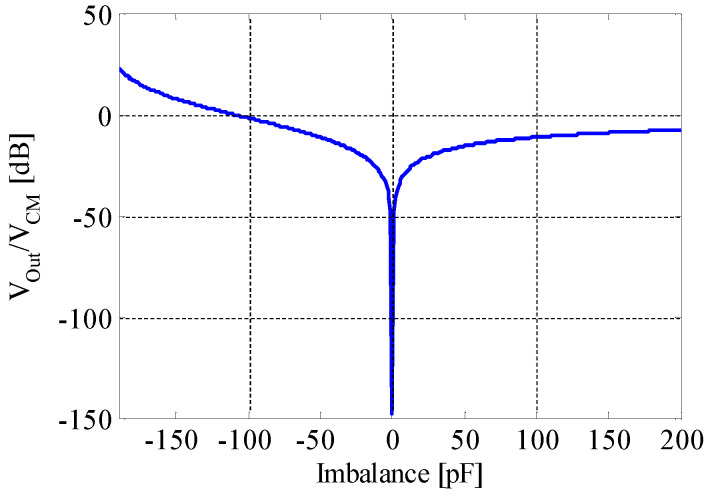
Simulated result of output voltage normalized to CM noise input for capacitance imbalance −200 pF to 200 pF (Table 1’s condition).

Figure 5 shows the simulated result of output voltage normalized to CM noise for capacitance imbalance (−200 pF to 200 pF), i.e., the value of ($C_{ea}$ – $C_{eb}$) in Figure 4. When using a noncontact circular electrode, the capacitance can be calculated from

(11) $$C=\varepsilon_{r}\varepsilon_{0}\frac{\pi r^{2}}{d}$$

Suppose that there is a stiff plate ECG noncontact electrode with a radius *r* of 2 cm, and the relative permittivity $\varepsilon_{r}$ of clothes is about 5 to 10. The thickness *d* between the human body and the electrode could be about 0.1 to 0.5 mm, depending on the clothes. With these parameters, the EBI capacitance could be 100 to 200 pF. Therefore, we assume that the capacitance of $C_{ea}$ and $C_{eb}$ are in hundred picofarad order. Ha et al. [21] also mentioned the approximate value of noncontact electrodes’ coupling capacitance as 1 pF to 10 nF, depending on their type and attachment conditions. From Figure 5, it can be seen that when the absolute value of imbalance increases, the noise output becomes larger. If $C_{ea}$ is near to 0 (which means that the attachment to human body is near to open), the CM noise increases severely. If the capacitances of $C_{ea}$ and $C_{eb}$ are matched exactly, $V_{out}$/$V_{CM}$ becomes −150 dB, which implies that the noise output is near to zero when there is no imbalance. Generally, the imbalance of noncontact EBI capacitance is tens of picofarads or larger [27].

### 3.2. $V_{out}$/$V_{CM}$ vs. CMRR

$V_{out}$/$V_{CM}$ is a simple parameter we use in this work to evaluate how much noise could be converted from an external interference CM voltage. $V_{out}$ is with respect to the circuit ground and $V_{CM}$ is with respect to the earth ground. It can be converted to the CMRR by using the following equations:

(12) $$CMRR=20log(\frac{A_{DM}}{A_{CM}})$$

In our simulation conditions, the effective CM voltage (with respect to the circuit ground) $V_{CMeff}$ and $V_{CM}$ has the following relation (when $V_{CM}$ is 1 V at 60 Hz):

(13) $$20log(\frac{V_{CMeff}}{V_{CM}})\approx -6\;\mathrm{dB}$$

Moreover, the common mode gain $A_{CM}$ in CMRR can be written as:

(14) $$A_{CM}=\frac{V_{out}}{V_{CMeff}}$$

Differential mode gain $A_{DM}$ is normally a fixed value 60 dB, which almost does not change in the simulation due to the high input impedance. Thus, we can obtain

(15) $$CMRR\approx 54\;\;\mathrm{dB}-20log\frac{V_{out}}{V_{CM}}$$

For example, when $V_{out}$/$V_{CM}$ is −150 dB, CMRR is about 204 dB. Generally, this value will be about 100 dB for an actual instrumentation amplifier at 60 Hz, because the error in the circuit elements could reduce the CMRR by about 40 to 60 dB. We use $V_{out}$/$V_{CM}$ as the main benchmark, because high total CMRR is not the main aim in this work.

### 3.3. “DTC Series”

**Figure 6:**
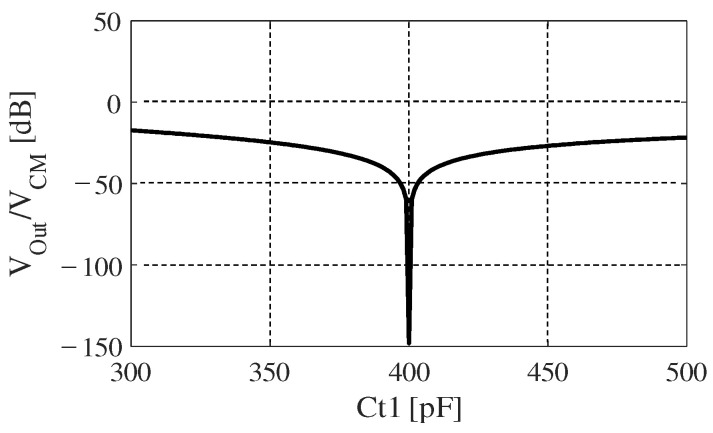
Simulated result of output voltage normalized to CM noise for “DTC Series” under Table 2’s condition.

Next, we perform another simulation to confirm the effectiveness of our proposal “DTC Series”. Table 2 shows the parameters we used to simulate the circuit elements in Figure 2. The other parameters are the same as we have shown in Table 1. Figure 6 shows the simulated result of output voltage normalized to CM noise for “DTC Series”. $C_{t1}$ is tuned from 300 pF to 500 pF, and we can see that $V_{out}$/$V_{CM}$ becomes −150 dB when $C_{t1}$ is 400 pF. At this point, capacitances of $C_{t1}$ and $C_{t2}$ compensate the imbalance between $C_{ea}$ and $C_{eb}$ exactly, such that the CM noise is extremely reduced. Comparing this result with Figure 5, this circuit can drive down the $V_{CM}$ by more than 20 dB if the imbalance is cancelled to below 20 pF.

### 3.4. “DTC Bypath”

**Figure 7:**
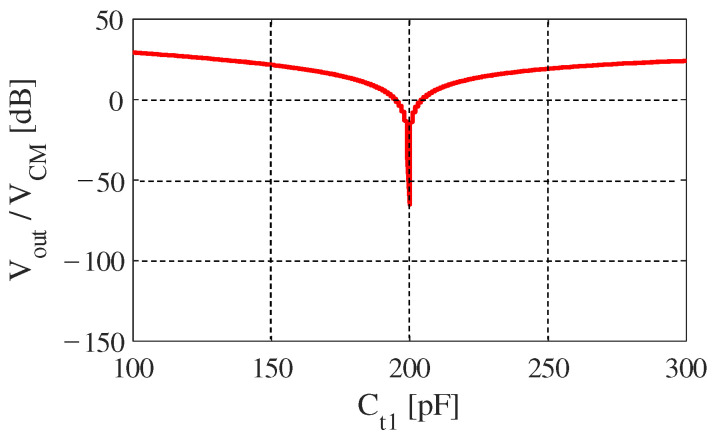
Simulated result of output voltage normalized to CM noise for “DTC Bypath” under Table 3’s condition.

We performed a simulation in almost the same way on “DTC Bypath”. The parameters of the circuit components in this simulation are shown in Table 3, and the results are shown in Figure 7. Because $C_{t2}$ is 400 pF, by calculation we can know that $C_{t1}$ should be about 199.5 pF to meet the relation in (9), which matches the simulated result in Figure 7. At its lowest point, $V_{out}$/$V_{CM}$ becomes about −70 dB. By comparing the two results in Figure 6 and Figure 7, we can see that generally “DTC Series” works better than “DTC Bypath” in the same conditions. This is because “DTC Bypath” did not completely cancel the imbalance like “DTC Series” in this simulation. According to (9), if we want to completely cancel the imbalance, $C_{t1}$ and $C_{t2}$ should be two very large values. However, we cannot use unlimitedly large capacitors in the “DTC Bypath” design because it increases current flow and reduces the gain of differential mode signal voltage. For this reason, we made $C_{t2}$ have the same order with $C_{eb}$ and swept $C_{t1}$, which is not a perfect cancellation, but a reasonable specification to evaluate this design.

### 3.5. Estimating the Practical Tunability Step of the DTCs

In the last two sections, we used rounded values for $C_{ea}$ and $C_{eb}$. In a realistic case, these capacitances could take any value in a certain range. This may cause a gap between the simulated result and the actual performance. However, there are some facts that can help us estimate the tunability step of the DTCs, such that we can fill this gap.

The first fact is that the capacitance of a noncontact electrode generally varies in hundreds or tens of picofarad order, less than 1 nF. In this range, the two EBI capacitances are generally in the same order, such that the noise rejection is determined by the absolute value of the imbalance between them. From (3), we can also know this fact by using the actual factors: the input impedance $Z_{in}$ is high enough to eliminate the influence of $C_{ea}$ or $C_{eb}$ in the denominator, and the output voltage be determined by the absolute value of the imbalance. Because of this fact, our simulation at the rounded point can be persuasive.

The second fact is that the actual accuracy of the adjustable capacitance step can be limited by many other factors. In our idea, basically the precision of the DTC cannot be higher than picofarads order. Stray capacitances and other factors can bring varies of errors to the circuit, which is also about several picofarads. Because of the errors, it is not very practical to use large $C_{t1}$ and $C_{t2}$ to match (9), making the reasonable tunability step of “DTC Bypath” also stay in picofarads order.

Considering these two facts, we can know that the reasonable tunability step of the DTCs is in picofarads order.

### 3.6. Frequency Characteristic

**Figure 8:**
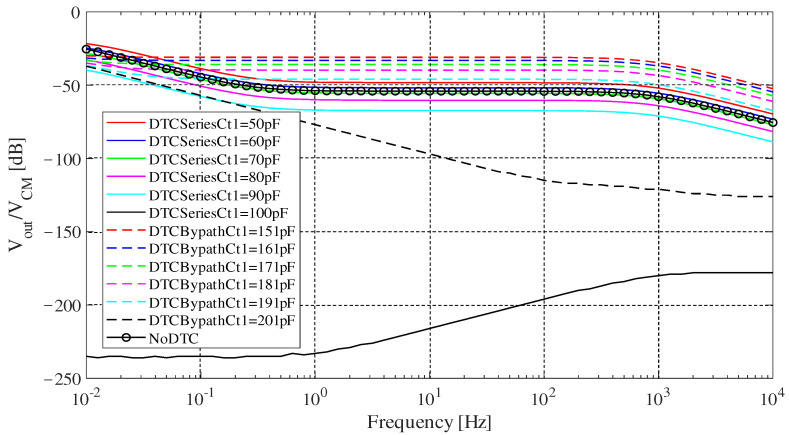
Simulated result of small signal AC sweep under Table 4’s condition. The solid lines show results for “DTC Series”, the dashed lines show results for “DTC Bypath”, “NoDTC” shows the results of no imbalance cancellation.

In order to investigate the frequency characteristic of each design, small signal AC (alternating current) sweep was conducted and the results are shown in Figure 8. The frequency ranges from 0.01 Hz to 10 KHz. The imbalance between $C_{ea}$ and $C_{eb}$ was set to 100 pF. Parameters of the simulated circuit components are shown in Table 4. $C_{t2}$ was fixed at a certain value and $C_{t1}$ in “DTC Series” and “DTC Bypath” were tuned by every 10 pF until they achieve a best case in rejecting the noise. From Figure 8, we can see that when $C_{t1}$ is tuned, performance of both circuits get better generally, and “DTC Series” basically works better than “DTC Bypath” when the frequency is larger than 0.1 Hz. Figure 8 also showed that in most frequency ranges, “DTC Bypath” could even make it worse than “NoDTC” when $C_{t1}$ is smaller than 10.

Comparing the best cases, i.e., “DTCSeriesCt1 = 100” and “DTCBypathCt1 = 201”, $V_{out}$/$V_{CM}$ of “DTC Bypath” remained at a high level when the frequency was lower than 1 Hz, while the noise reduction effect of “DTC Series” performed better than “DTC Bypath” by more than 100 dB at 1 Hz. When the frequency increased, the noise reduction effect of “DTC Series” became worse. Most artifacts are at low frequency (below 100 Hz). In conclusion, “DTC Series” generally works better than “DTC Bypath” in the frequency range of biopotential signals. Besides, it should be noted that the frequency response of an actual biosignal acquisition circuit can affect the result.

## 4. Imbalance Detection Strategy and Experimental Evaluations

In last section, we have shown that “DTC Series” had a better performance, thus, we choose this design and perform experimental evaluations on it. In this section, we first explain our strategy to detect the imbalance of EBI impedance. After that, we present our experimental evaluation on imbalance detection and CM noise reduction effect. Earlier, we showed that if the imbalance is cancelled, the noise output of a common-mode interference voltage will be rejected to a very low level. In order to realize this goal, we must know how much imbalance exists at the EBI.

**Figure 9:**
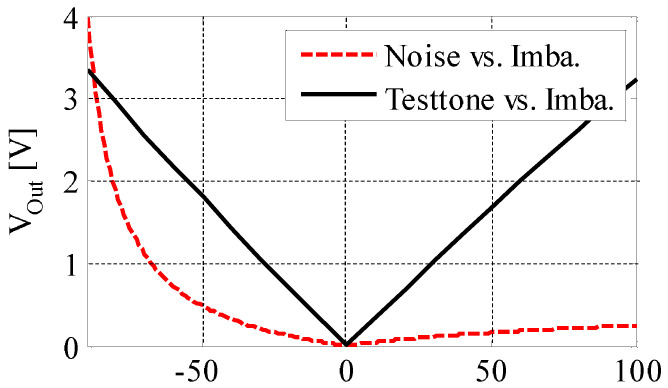
Correlation between the test signal and the electrode—body interface (EBI) imbalance of conventional method and our circuit design. Red dashed curve: with the conventional method (input voltage vs. the imbalance); black curve: with our design (test tone vs. the imbalance).

In [22], a method that uses the two voltage inputs of the differential amplifier was mentioned. However, this method has three disadvantages. The first one is that it receives many interferences. For example, when the biopotential voltage is at a high level, it may cause significant errors in deciding if the CM noise level is higher than the threshold or not. Our design solves this problem by employing an injection sine wave signal. The second one is that it does not have good linearity for capacitance. Figure 9 shows the difference between our approach and the conventional detection method. With good linearity, the imbalance cancellation process could be accomplished more preciously and simply.

The third disadvantage of it is that the input voltage is difficult to filter. In the biopotential frequency range of about 0.01 Hz to 1 kHz, the CM voltage component can appear at any frequency. The conventional method cannot find out if the input voltage is really related to the CM noise source. On the other hand, our design employs a sine wave of 1 kHz, making it easy to be filtered and improve the precision of imbalance monitoring.

**Figure 10:**
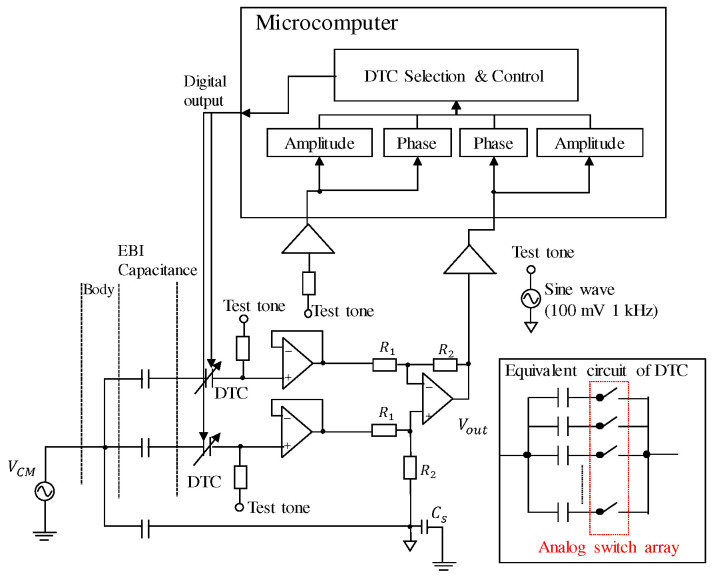
Configuration of the imbalance cancellation system with a microcomputer.

Figure 10 shows the configuration of our system to detect and cancel the imbalance. In order to monitor the variation of the imbalance continuously, we employed an injection signal, or what we call a “test tone” in Figure 10, which is a sine wave with a frequency of 1 kHz and an amplitude of 100 mV. The test tone is injected at the positive input of the buffer, passing a resistor of 100 kΩ. When the capacitance of EBI changes, output of this injection signal at $V_{out}$ follows, such that we can detect this change by reading this output. In order to figure out the exact value of the imbalance in picofarad order, we read in the amplitude and the phase information of the injection signal input and output. We can know which capacitance ($C_{ea}$ or $C_{eb}$) is larger by comparing the phase, because it reverses by 180 degree depending on whether $C_{ea}$ or $C_{eb}$ is larger. Moreover, the amplitude of the output tells us the absolute value of the imbalance.

Due to this, the 100 kΩ resistor and the capacitance of EBI and DTC forms a high-pass filter, which could cause a decline in signal gain. However, during practical use, a switch could be employed to cut off the connection at the place of 100 kOhms resistor, such that the biosignal would only be affected during the cancellation process. When the CM noise level is high, we cancel the imbalance, and turn off the switch after the noise is driven down, only remaining the DTCs in the front end. Rejecting the interference of the biosignal could also be an advantage during the imbalance detection progress.

**Figure 11:**
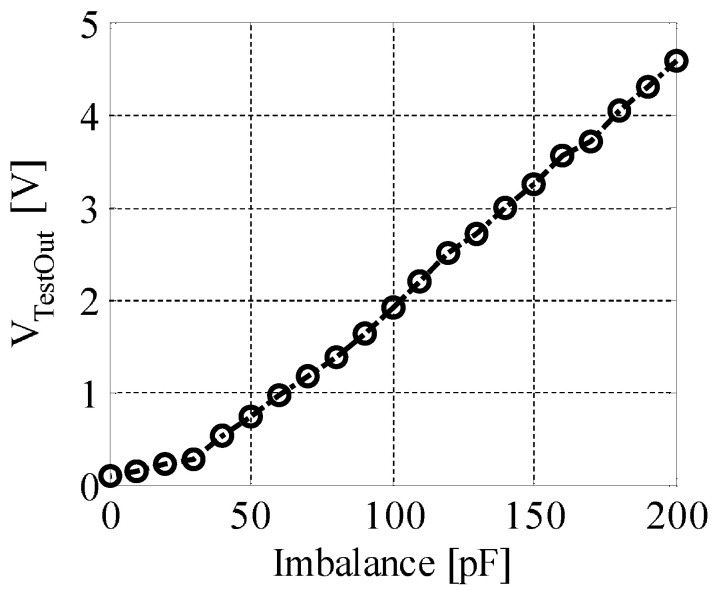
Correlation between the imbalance and the output of the injection signal (measured in experimental environments). When the absolute value of imbalance increases, the output voltage of test tone increases linearly.

Figure 11 shows the correlation between the imbalance and the output of the injection signal. From this figure, we can see that they are almost linearly related in our experimental environments. This property can also be confirmed by circuit simulation. In Figure 10, the equivalent circuit of our originally designed DTC is also presented. It consists of several channels of capacitors connected in parallel; each channel is controlled by one analog switch. For example, in ideal case, if we make the capacitance of each channel to be 1 pF, 2 pF, 4 pF, 8 pF, 16 pF, …, 512 pF, then the capacitance value can be binarily tuned in the range of 1 pF to 1 nF, by switching on or off each channel with a digital signal.

There are some other DTCs and the equivalent circuits can be drawn. The existing three types of DTCs include the RF (radio frequency) tuning micro-electro-mechanical system (MEMS), the barium strontium titanate (BST) device and the silicon on isolator (SOI)/silicon on sapphire (SOS) tuning devices. However, none of these three types of DTC can meet our demands because all of them only work rightly in radio frequency range. Other variable capacitor elements like a varicap diode cannot be linearly tuned with a digital signal. In summary, there was no other existing DTC that can meet all our demands: availability in biopotential frequency, big tunable range (hundreds of picofarads), good linearity, and digitally tunability. Therefore, we developed our original DTC. We employed the analog switch array, or multiplexer, to construct the structure shown in Figure 10. The analog switch array that we employed consists of MOSFETs (metal-oxide-semiconductor field-effect transistor). When they work as an analog switch array, the level of gate voltage controls the current flow between source and drain, realizing a digitally controlled switch-on or -off operation. Because we connected a capacitor in series on each channel and combined all the inputs or outputs respectively, the capacitance value increases when the channel switch is on or decreases when it is off. The multiplexer reads in digital data from the microcomputer such that it can be digitally tuned.

**Figure 12:**
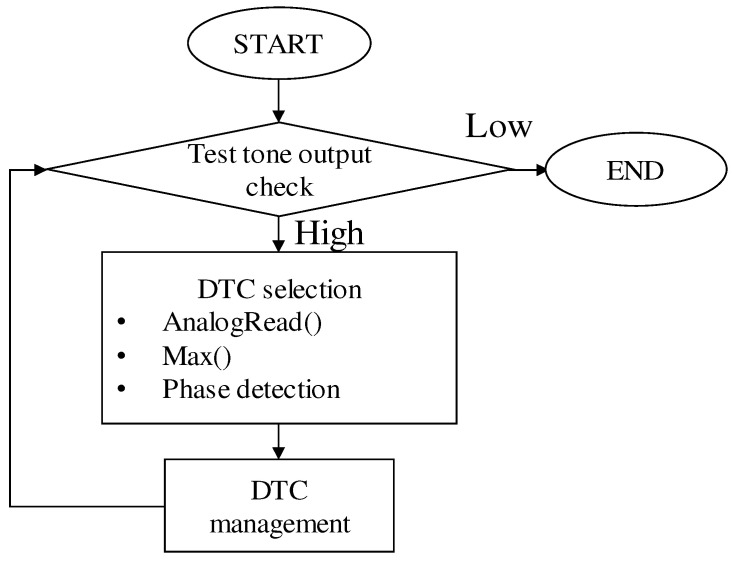
Flow chart of the imbalance detection and DTC management algorithm.

Figure 12 shows the flow chart of our imbalance detection and DTC management algorithm. A threshold value of test tone output was used to trigger the DTC tuning operation. When the output of test tone signal increases to a high level, the microcomputer reads in the input and output of the test tone, from the two ports of our biopotential acquisition circuit shown in Figure 10. After that, values of the phase and amplitude are employed to help tuning the DTCs, as we mentioned earlier. Lastly, we check the output of the test tone again to confirm that the imbalance cancellation is successful. This loop is supposed to be performed in the microcomputer continuously because we expect that the CMRR can be restored automatically during actual use.

To make the system realize fully automatic imbalance cancellation during noncontact biopotential, acquisition is always a goal. The system and the algorithm in the microcomputer can generally work automatically, but there are still some challenges in its optimization, especially in controlling the DTCs. In this work, presenting the value and potential of the approach is our focus.

**Figure 13:**
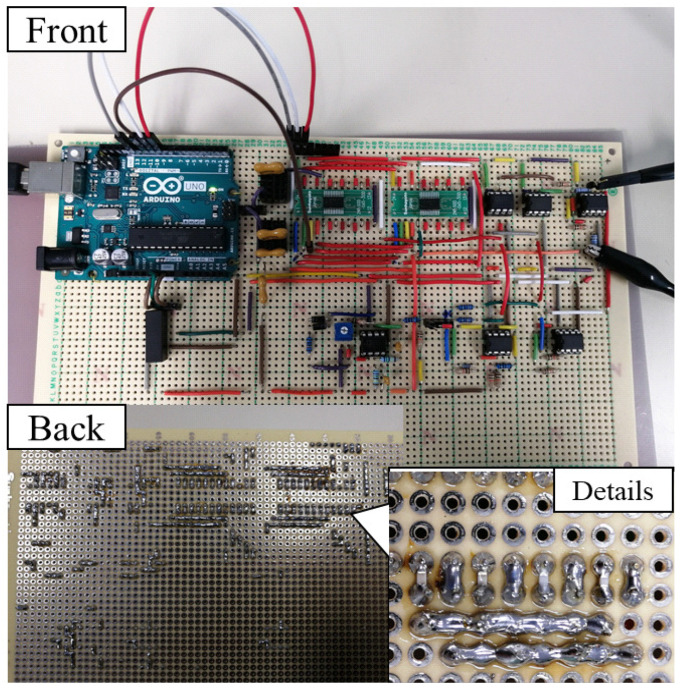
Evaluation circuit on a universal board. We use two sets of ceramic capacitors to simulate the EBI capacitance. The chip capacitors for constructing the DTC are soldered on the back side of the board.

Figure 13 is a picture of our evaluation board. We constructed the system shown in Figure 10 on a universal board to experimentally evaluate the performance of our design. We use two sets of ceramic capacitors to simulate the EBI capacitance. A coupling voltage of 1 V, 60 Hz (also a sine wave, but different from the 100 mV, 1 kHz test tone) as a pseudo CM noise source $V_{CM}$, which was generated with a function generator. A Wien bridge oscillator circuit was developed to provide the 1 kHz sine wave injection signal. The chip capacitors in the DTCs are soldered on the backside of this board. We first used ordinary capacitors like ceramic capacitors or mica capacitors, instead of DTCs to exactly cancel the imbalance, to see how much the CM noise is reduced when our design works with no need to worry about the digitally tuning part.

**Figure 14:**
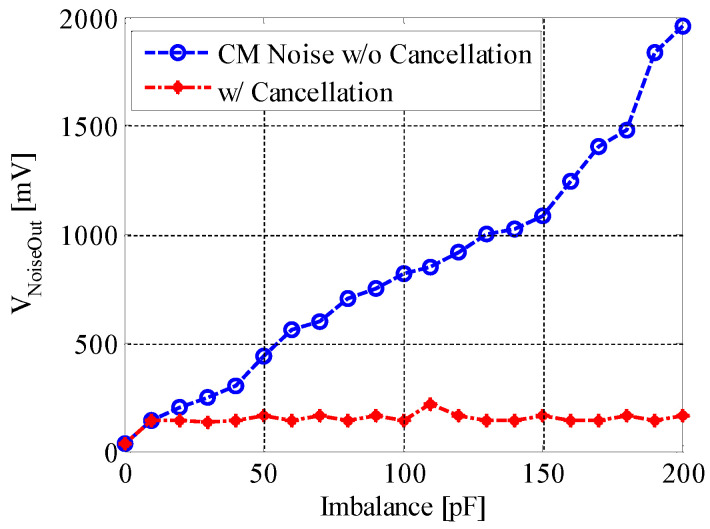
Noise reduction efficacy of our approach. The noise source is a 1 V, 60 Hz sine wave. The blue circled line is the CM noise output when the imbalance is forced in the capacitors emulating the EBIs; the red dotted line is the result when the imbalance is overcame by means of appropriate selection of ordinary capacitors (manually).

Figure 14 shows the CM noise reduction efficacy when ordinary capacitors like ceramic capacitors or mica capacitors are used to cancel the imbalance. We can see from this figure that when the imbalance is cancelled, the noise output from our evaluation board remains at a low level, around 60 mV. The noise level can be driven down by about 30 dB when the imbalance is as large as 200 pF. This result generally matches our simulation result, and the remaining noise voltage could be from the other parts of the circuit, like the amplifiers.

**Figure 15:**
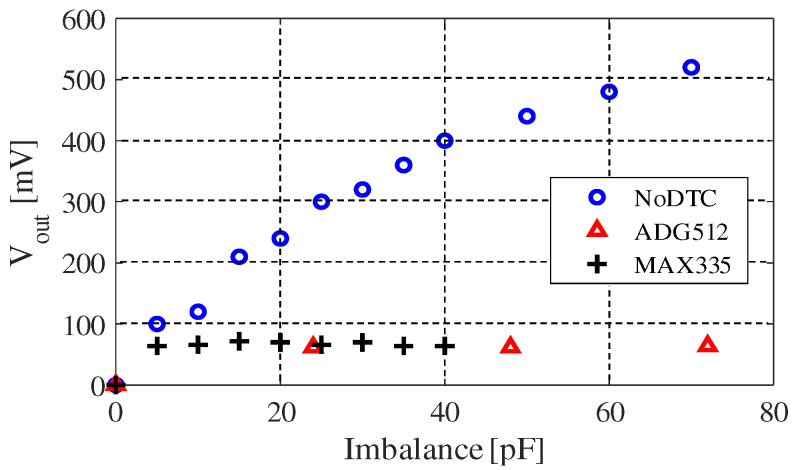
Experimental result of noise reduction efficacy using DTCs. ADG512: 4 tunable steps, about 24 pF for each step; MAX335: 8 tunable steps, about 6 to 8 pF for each step.

Figure 15 shows the experimental evaluation result of our originally constructed DTC. We have tried several kinds of analog switch array elements, among them ADG512 (analog devices) and MAX335EUG+ (maxim-integrated) are chosen because their features fit our demands best. Parameters of the two chips are shown in Table 5. They can both run with ±5 V voltage supply, and the channel number of ADG512 is 4 while MAX335EUG+ has 8 channels. The channel on-capacitance and channel off-capacitance are two important parameters, because during our test we found that they decide the equivalent capacitance of each channel. For example, the tuning step of the DTC using the ADG512 is about 24 pF, which is near to the difference between the channel on-capacitance and channel off-capacitance. The reason why it is smaller than (35–24 pF) is because we connected a capacitor in serial with it, to make the circuit more stable. In a word, the structure of CMOS limited its tuning step range.

From Figure 15, we can see that the equivalent capacitance of each channel is about 24 pF when we use the ADG512 chip. The tunable range of DTC using ADG512 is up to 96 pF, approximately. However, due to this, we used one of its channels as a switch (no capacitor connected to this channel), making the actual upper limit about 75 pF (approximately 3 × 24 pF). This value can change due to the circuit implementation and the chip capacitor we connected to it. On the other hand, MAX335EUG+ has 8 channels but the channel on-capacitance is about 8 pF. This makes the tunable range of our MAX335EUG+ version of DTC only able to be tuned up to around 50 pF. Because we used one of its channels as a switch, the actual upper limit is about 42 pF (7 × 6 pF). According to our measurement, the equivalent capacitance of each channel is about 6 to 8 pF, which was hard to be exactly measured because it is quite a small value. Moreover, we can only give the results in tens of picofarads order in Figure 15 in the same reason.

The actual performance of our approach could still be significantly improved when better DTCs are employed in the circuit. The tuning step of the digitally tunable capacitors (DTCs) has certain influences on the accuracy of this technique. Approximately the drop of noise reduction caused by the mismatches between the EBI capacitances and the DTCs can be estimated from Figure 14. For example, if the tuning step of the DTC is 24 pF, the noise output rises by about 100 mV in the worst case.

## 5. Discussion and Summary

Noncontact/capacitive biopotential sensing technology has a great future in medical treatment, healthcare and new applications with wearable technology. The disadvantages of the contact electrodes, about comfort and longevity, can be solved by insulated biopotential sensing technology, but the common-mode noise rejection is still a challenging task for noncontact electrodes. In this work, we have presented an approach to reduce the CM noise output of a noncontact biopotential acquisition circuit (analog front end), which works through cancelling the imbalance of EBI impedance with DTCs. At first, we provided a circuit model of noncontact biopotential acquisition, and analyzed our proposal through calculations and simulations. We clarified how much influence an imbalance in the EBI could have for a typical biopotential acquisition circuit, showing that our proposal is feasible by simulation results. We also made a comparison with another circuit design, showing the frequency sweep result of both circuits. Based on the simulation results, the first proposal that we outlined in the “DTC Series” was our last choice. Secondly, we proposed a design that uses a microcomputer together with originally designed DTC to cancel the imbalance. An injection signal was employed to detect and monitor the impedance change of the EBI, such that we can allow the microcomputer control the DTCs accordingly. The performance of this design was evaluated on a circuit board in experimental environments. When ordinary capacitors like ceramic capacitors or mica capacitors are used for evaluation, the result showed that the CM noise level can be driven down by about 30 dB when there is an imbalance of 200 pF, which matches our simulations quite well.

It is a fact that some existing tunable capacitor elements have almost the same design as our DTC. However, the novelty of our design still lies in its advantages of realizing good digital tunability and availability in our EBI imbalance cancellation circuit. In the future, we expect that new design of DTC with better linearity, larger capacitance variation and better tunability can improve the performance of this design. Embedding a tunable capacitor to make a new type of noncontact electrode is also in our vision.

Aside from this, the analog-front-end of our design have very large input impedance (more than 160 MΩ when OP07 is used) and low current. The main energy cost is from the microcomputer, whose power consumption can be driven down to as low as 23 µA with 5V power supply, in power-down sleep mode. DC power supplies of the analog elements are all provided by the microcomputer.

In summary, we presented a novel approach to reduce CM noise for noncontact biopotential acquisition, which could help support long-term, comfort and precise applications of wearable devices, human—computer interface or IoT (Internet of Things).

## Figures and Tables

**Table 1:** Parameters of circuit components in Figure 4.

Parameter	Value	Parameter	Value
Frequency	60 Hz	$V_{CM}$	1 V
$C_{ea}$	1 pF to 400 pF	$R_{1}$	10 kΩ
$C_{eb}$	200 pF	$R_{2}$	10 MΩ
$C_{eg}$	200 pF	$C_{s}$	200 pF

**Table 2:** Parameters of the simulated circuit components in Figure 2.

Parameter	Value	Parameter	Value
Frequency	60 Hz	$V_{CM}$	1 V
$C_{ea}$	200 pF	$C_{t1}$	300 pF to 500 pF
$C_{eb}$	400 pF	$C_{t2}$	200 pF

**Table 3:** The parameters of the simulated circuit components in Figure 3.

Parameter	Value	Parameter	Value
Frequency	60 Hz	$V_{CM}$	1 V
$C_{ea}$	200 pF	$C_{t1}$	100 pF to 300 pF
$C_{eb}$	400 pF	$C_{t2}$	400 pF

**Table 4:** Parameters of the circuit components for the alternating current (AC) sweep.

Parameter	Value
$C_{ea}$	200 pF
$C_{eb}$	100 pF
$C_{t1}$ (“DTC Series”)	50 to 100 pF
$C_{t2}$ (“DTC Series”)	200 pF
$C_{t1}$ (“DTC Bypath”)	151 to 201 pF
$C_{t2}$ (“DTC Bypath”)	100 pF

**Table 5:** Parameters of ADG512 and MAX335EUG+

	ADG512	MAX335EUG+
Voltage Supply	±5 V	±5 V
Number of Channels	4	8
Channel On-Capacitance	35 pF	8 pF
Channel Off-Capacitance	9 pF	2 pF

## Abbreviations

The following abbreviations are used in this manuscript:

CM	Common Mode
CMRR	Common Mode Rejection Ratio
ECG	Electrocardiogram
EEG	Electroencephalogram
EOG	Electrooculogram
DTC	Digitally Tunable Capacitor
AC	Alternating Current
DC	Direct Current
OP-AMP	Operational Amplifier
